# Cooperative hand movements: task‐dependent modulation of ipsi‐ and contralateral cortical control

**DOI:** 10.14814/phy2.13581

**Published:** 2018-05-15

**Authors:** Miriam Schrafl‐Altermatt, Christopher S. Easthope

**Affiliations:** ^1^ Spinal Cord Injury Center Balgrist University Hospital Zurich Switzerland; ^2^ Neural Control of Movement Laboratory Department of Health Sciences and Technology ETH Zurich Switzerland

**Keywords:** electromyographic reflex responses, electrophysiology, neural coupling, somatosensory evoked potentials

## Abstract

Cooperative hand movements are known to be controlled by a task‐specific neural coupling associated with an involvement of the respective ipsilateral hemispheres. The aim of this study was to explore in how far this neural control applies to and is modulated during various, fine and gross, cooperative hand movements required during activities of daily living. Somatosensory evoked potentials and contralateral electromyographic reflex responses to unilateral ulnar nerve stimulation were simultaneously recorded in healthy participants during three different cooperative hand movement tasks and a resting condition. Amplitude ratio (ipsi‐/contralateral) of the somatosensory evoked potentials, which is a measure for the involvement of the ipsilateral hemisphere in movement control, was higher in all three movement tasks compared to resting. This ratio was highest during the fine cooperative movement studied here. Contralateral reflex responses, as a measure for the functional coupling of the arms, were elicited following stimulation of both arms during gross cooperative movements. However, such a response could only be elicited in the dominant arm during fine movement. It is concluded that the neural coupling and thus enhancement of ipsilateral cortical control is preserved through different cooperative hand movement tasks, independently whether fine or gross motor tasks are performed. However, modulation of cortical control can be observed as ipsilateral cortical control is stronger during fine movements and functional coupling of the arms more focused to the dominant hand compared to gross cooperative tasks.

## Introduction

Recently, a task‐specific neural control of cooperative hand movements has been described in healthy (Schrafl‐Altermatt and Dietz 2014; Dietz et al. 2015) and poststroke participants (Schrafl‐Altermatt and Dietz 2016a,b). During cooperative hand movements, both hands are linked over a kinematic chain. The force applied by one hand is counteracted by the other one in order to achieve the movement goal, for example, opening a bottle. In contrast to other bimanual movements (Debaere et al. 2001; Gerloff and Andres 2002; Swinnen 2002; Carson 2005), cooperative hand movements are controlled by a neural coupling mechanism. This mechanism is reflected electrophysiologically in bilateral electromyographic (EMG) reflex responses in activated forearm muscles to unilateral arm nerve stimulation (Dietz et al. 2015; Schrafl‐Altermatt and Dietz 2016a) and a higher ipsi‐ to contralateral amplitude ratio of somatosensory evoked potentials (SSEPs) (Schrafl‐Altermatt and Dietz 2014, 2016b) and by imaging (fMRI) in a stronger activation of the bilateral secondary somatosensory (S2) cortical areas during cooperative hand movements when compared to bimanual noncooperative tasks (Dietz et al. 2015). This indicates a strong involvement of the ipsilateral hemisphere in this control.

All studies investigating the neural coupling of cooperative hand movements have so far focused only on the analysis of the bottle opening/closing task although many tasks required during activities of daily living (ADLs) comprise cooperative hand movements. It is known that neural interlimb coupling described for locomotor movements can be task dependently modulated (Carpinella et al. 2010; Kloter et al. 2011; Kloter and Dietz 2012) while basic characteristics of the coupling remain preserved (Wannier et al. 2001).

The main goal of this study was to investigate the modulation of neural coupling during different cooperative hand movements – one being a small (screwing a bolt into a nut) and the other one being a larger movement (sawing wood) – and to compare these with the “opening a bottle” task. To avoid differential effects on SSEPs and reflex modulation driven by adaption or learning, we employed a novel technique in simultaneously recording SSEPs and EMG reflex responses following unilateral arm nerve stimulation. This allows for direct comparison of these two aspects of the neural coupling mechanism. SSEPs are recorded over the primary somatosensory cortical areas (S1). Based on previous results, it is suggested that activation of S2 during cooperative hand movements upregulates the excitability of ipsilateral S1. Thus, changes in perception of the movements should influence S1 SSEPs. EMG reflex responses, however, are additionally influenced by movement parameters. Therefore, it is hypothesized that both contralateral EMG reflex responses and ipsilateral SSEPs are modulated by the different tasks in a differential way, while the basic mechanism of neural coupling remains preserved.

## Methods

The study conformed to the declaration of Helsinki and was approved by the local ethics committee (Kantonale Ethikkommission Zürich). All participants gave their written informed consent prior to enrolment. Fifteen healthy adults (9 females) with a mean age of 28 ± 3.3 years and a mean height of 171.9 ± 7.8 cm participated in this study.

### General procedures

**Figure 1:**
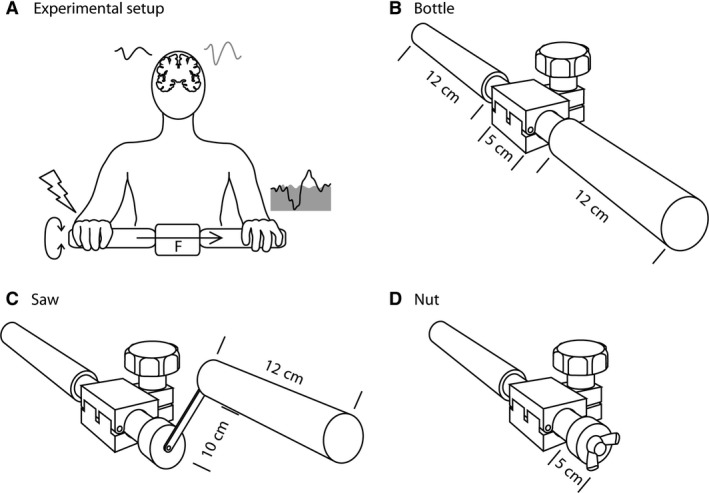
Experimental setup and study device. (A) During the experiment, the force generated by the rotational movements against a given torque by the dominant hand was transferred to the opposite handle of the device and had to be compensated for by the exertion of a corresponding isometric torque by the nondominant hand. During the motor tasks and the resting condition, the ulnar nerve of either the dominant or the nondominant side was electrically stimulated. Somatosensory evoked potentials over both hemispheres and electromyographic reflex responses of the forearm muscles were recorded simultaneously (see Methods). Cooperative task conditions (B–D): The study device comprised of a one handle fixed to a shoe‐type brake and was connected with an exchangeable handle. The resistance for counteractive rotation of the two handles could be adapted by tightening and loosening the screw of the brake. (B) For the bottle condition a cylindrical handle was applied. (C) For the saw condition, a cylindrical offset‐handle was used and (D) for the nut condition a butterfly nut mounted on a short cylinder.

The protocol comprised simultaneous recordings of SSEPs over both hemispheres and EMG reflex responses in forearm extensor and flexor muscles of both sides to unilateral ulnar nerve stimulation (Fig. 1A) during a resting condition and during three cooperative hand movement tasks. In all three movement tasks, a device similar to the one described previously was used (Schrafl‐Altermatt and Dietz 2014, 2016a,b; Dietz et al. 2015). Figure 1B‐D shows the device with exchangeable handles that was used to perform the three cooperative movement tasks. The exchangeable handles for the dominant hand mimicked three daily living tasks requiring cooperative hand movements. The device shown in Figure 1B (bottle) matches the condition for reflex studies performed previously in healthy (Dietz et al. 2015) and poststroke participants (Schrafl‐Altermatt and Dietz 2016a) as well as the condition used in SSEP studies in healthy (Schrafl‐Altermatt and Dietz 2014) and poststroke (Schrafl‐Altermatt and Dietz 2016b) participants. It mimics a bottle opening and closing movement that is performed by wrist flexion and extension movements. The saw condition (Fig. 1C) represents a gross cooperative task involving elbow and shoulder flexion and extension movements. The third “nut” condition (Fig. 1D), reflects a small precise cooperative movement task, requiring a pinch grip of thumb and index finger and pro‐ and supination movement of the forearm. The handle held by the nondominant hand was the same for all movement conditions. It was stabilized in order to counteract the movements performed by the dominant hand by exerting a corresponding opposite isometric torque. The resistance for counteractive rotations of the handles was set at 1 Nm, 1.5 Nm, and 0.5 Nm for bottle, saw, and nut, respectively.

Participants were instructed to perform the movements with a frequency of 0.67 Hz (i.e., one movement cycle in 1.5 sec). They were lying in a supine position during all conditions with closed eyes and instructed to keep their mouths slightly open, not to talk and to avoid swallowing in order to minimize artifacts in the EEG recordings. Each condition was performed for about 3 min in a randomized order. During each condition the ulnar nerve was stimulated first either on the dominant moving (Fig. 1A) or on the nondominant, holding (not shown) arm followed by stimulation of the other side. Thus, each experiment consisted overall of eight recording blocks (three tasks and the resting condition).

In one participant, EEG was measured in three additional nonmoving conditions. The participant was lying in the same position, holding the device with one of the handles in an endpoint position (bottle: right wrist extended, left wrist flexed; saw: right hand up; nut: right arm supinated). The handle was changed for every of these additional nonmoving conditions.

### Ulnar nerve stimulation for evoking reflex responses and SSEP's

The ulnar nerve of each arm (in random order) was stimulated 15 times every three to ten seconds during each of the four conditions using a KeyPoint XP device (Medtronic A/S, Skovlunde, Denmark) through self‐adhesive surface electrodes (5.96 mm^2^, CareFusion, Middleton, Wi, U.S.) which were placed over both wrists with an inter‐electrode distance of 2 cm. Stimulation intensity (SI) of the ulnar nerve was set at 0.5 mA above motor threshold (MT), that is, lowest intensity resulting in visible twitching of the M. abductor digiti minimi. Additionally, sensory thresholds (ST) were registered. ST is defined as lowest intensity at which two out of two stimulations are perceived. Stimulations consisted of a 333 Hz train of four biphasic pulses of 1 ms duration resulting in a total stimulus duration of 10 msec. This stimulation protocol has been chosen to elicit both SSEPs as well as EMG reflex responses. SSEPs are usually evoked by single stimuli of short durations with frequencies between 1 and 5 Hz (Fujii et al. 1994). EMG reflex responses, on the other hand, are usually elicited by trains of 4 to 8 pulses at 200 to 300 Hz with total stimulation lengths of 20 to 40 msec which are delivered at randomized frequencies with at least 4 sec between two stimulations to minimize habituation (Zehr and Kido 2001; Hubli et al. 2013; Schrafl‐Altermatt and Dietz 2016a). In order to elicit both SSEPs and reflex responses simultaneously, a new stimulation protocol had be elaborated. The requirements included (1) stimulation length of less than 10 msec due to the expected N20 peak in the SSEPS that needed to be clearly separated from the stimulation artifact; (2) train of at least 4 biphasic pulses to consistently evoke reflex responses; (3) ability to reliably record SSEPs with only 10 to 20 repetitions as the interstimulus interval needed to be at least 4 sec and the quality of the movement could only be ensured over a few minutes. Five dummy stimulations (intensity set to 0 mA) were released before and five after the 15 active stimulations in order to calculate background EMG activity.

### EEG recordings

Bilateral cortical SSEPs were recorded by KeyPoint XP (Medtronic A/S, Skovlunde, Denmark) through needle‐electrodes (12 mm, Spes Medica S.r.l., Battipaglia, Italy) placed over Fz (as reference), C3 and C4. C3 and C4 lie over the hand areas of S1. Signals were recorded with a frequency of 12,000 Hz and band‐pass filtered between 6000 Hz and 1 Hz. After the recordings, all signals were further analyzed with Soleasy (ALEA Solutions GmbH Software & Instrumentation, Zurich, Switzerland). A Butterworth band‐stop filter (45‐55 Hz) was applied to exclude any possible 50 Hz noise from the EEG signal. All recordings per side and condition were averaged for every participant before calculation of latencies and amplitudes. Latencies of the potentials were automatically set at minima between 17 msec and 25 msec (N20) and maxima between 21 msec and 29 msec (P25). Latencies were also visually verified and the time window for automatic latency calculation adjusted if needed. Amplitudes of the potentials were calculated as difference between N20 and P25. The ratio of the potentials of both sides was calculated by the division of ipsilateral by contralateral SSEP amplitudes. The time scale was normalized for illustrating purposes by setting the individual N20 peak of every trace to zero before calculation of grand averages.

### EMG recordings

EMG activity of wrist flexor (flexor carpi ulnaris) and extensor (extensor carpi radialis) muscles of both forearms was recorded using dual surface electrodes (Dual Electrodes #272S, Spacing 2 cm, Noraxon, Scottsdale, AZ). Signals were sampled at 1500 Hz and recorded using a wireless EMG system (Noraxon, Scottsdale, AZ). Recordings were further processed using Soleasy (ALEA Solutions GmbH Software & Instrumentation, Zurich, Switzerland). EMG signals were offset corrected, rectified and band‐stop filtered (45–55 Hz). Root mean squares (RMS) of EMG activity were calculated for each trial for the time window of 50–200 msec after stimulation onset. All active and all dummy trials were each averaged for every condition and each stimulated side. Latencies of the N2/P2 complex were calculated as minima between 70 and 130 msec and maxima between 100 and 200 msec. These time windows were adjusted if needed. The duration of EMG reflex responses was calculated between the point of time when the EMG signal was below the standard deviation (SD) of the prereflex background EMG activity (PA; 40–50 msec after stimulation onset) for at least five consecutive milliseconds and the point of time when the EMG signal was back in the range of mean PA ± SD for at least five consecutive milliseconds after having exceeded PA + SD for at least five consecutive milliseconds. If these criteria were not met during a poststimulus trace, the trace was classified as a missing reflex response. Percentages of participants showing reflex responses were calculated for every condition and stimulated side and defined as presence of a reflex response.

### Motion capture

A kinematic analysis was performed in one of the participants in order to illustrate the different movement tasks. Reflective markers (14 mm) were attached to anatomical reference positions on the hands, arms and trunk of the participant as well as on the device with at least 3 markers defining each segment. Marker positions were recorded in Nexus 2.3 (Vicon Motion Systems Ltd., Oxford, UK) from 10 infrared cameras (T‐Series, Vicon) at 200 Hz. Segment positions were calculated using an optimal common shape approach (Taylor et al. 2010). Euler angles were obtained for each joint (Ehrig et al. 2007) in all three planes in a hierarchical manner. Trials were then segmented for cyclic motion, and a grand mean was calculated. The kinematic trajectories were recorded in synchronized fashion with EMG (1500 Hz, same equipment as described above) from the biceps and triceps muscles as well as the wrist extensor and flexor. The kinematic signals were only used for visualization purposes of the movement cycles in the three cooperative tasks. Therefore, except of the segmenting and averaging of the raw signal, no further signal processing was applied.

### Statistics

Statistics were calculated using IBM SPSS Statistics 19 (Armonk, New York, U.S.). Differences between RMS of background EMG and EMG following stimulation were calculated with paired t‐tests. Differences in EMG reflex duration, SSEP amplitude ratios and latencies of both reflexes and SSEPs were calculated using general linear models with post hoc t‐tests with Bonferroni corrections. Equivalency of amplitudes and latencies was calculated with two one‐sided t‐tests (TOST procedure).

## Results

All participants tolerated the experimental procedures well and perceived the stimulations at 150% MT as non‐noxious but clearly perceptible. Results are given as mean values plus/minus standard deviation. Intensities at ST, MT and for stimulation did not differ between the two hands (Moving hand: ST = 0.85 ± 0.24 mA, MT = 3.19 ± 0.88 mA, SI = 4.52 ± 1.24 mA; holding hand: ST=0.81 ± 0.24 mA, MT=3 ± 0.74 mA, SI=4.27 ± 1.14 mA). EMG reflex responses were most prominent in wrist extensors. The less pronounced responses in the flexors, showed modulation patterns which did not differ to those observed in the extensors. For simplification we only show traces and analyses of the EMG from the extensor muscles. Contralateral SSEP amplitudes were equivalent in all movement tasks and stimulation sides which indicates that the stimulation intensity reaching the nerve was equivalent in all conditions.

### Contralateral EMG reflex responses

**Figure 2:**
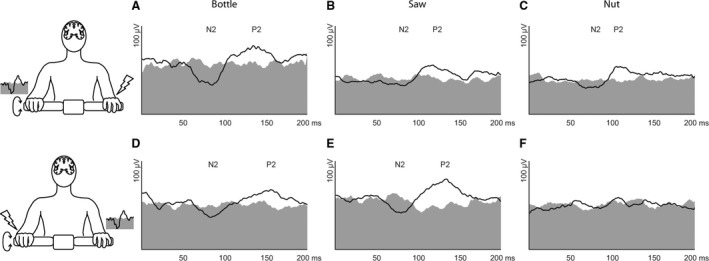
Electromyographic (EMG) reflex responses: Grand averages. Grand averages from all (*n* = 15) participants of the EMG recordings in the contralateral wrist extensors following stimulation of the holding (A–C) and the moving (D–F) hand are shown in black for the three cooperative movement conditions, that is, bottle (A, D), saw (B, E) and nut (C,F). Background EMG activity is displayed in gray. The typical second reflex component composed of a negativity (N2) and a positivity (P2) was elicited in all conditions but the nut task when the moving hand was stimulated.

**Figure 3:**
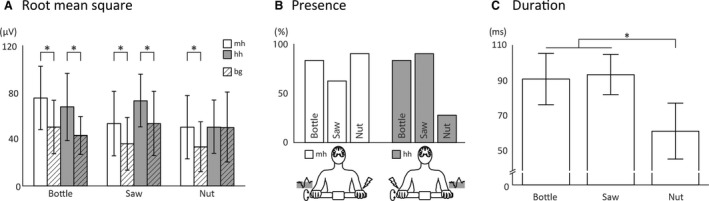
Electromyographic (EMG) reflex responses: Quantitative analyses. (A) The root mean square values calculated over a time window of 50 to 200 msec after stimulation onset are displayed for the active (reflex responses; nondashed bars) and dummy (background EMG; dashed bars) stimulations for all three task conditions. (B) The percentage of participants showing detecTable (for definition see Methods) reflex responses in the three task condition. (C) The mean duration of the reflex responses are shown for gross (bottle and saw) and fine (nut) cooperative hand movements. mh: reflex responses in the moving hand following stimulation of the holding hand; hh: reflex responses in the holding hand following stimulation of the moving hand; bg: background activity following dummy stimulation; error bars: standard deviation; *P < 0.05.

Figure 2 shows the grand averages of contralateral reflex responses in the wrist extensor muscle. In all three movement conditions a contralateral reflex response was elicited in the moving hand by ulnar nerve stimulation of the holding hand (Fig. 2A–C). However, stimulation of the moving hand elicited EMG reflexes in the contralateral holding hand during the bottle and saw tasks but not in the nut condition. This is also reflected in the RMS analysis (Fig. 3A) where significant differences in RMS between reflexes and background EMG are shown for all conditions in both hands except for the holding hand in the nut condition. Latencies in contralateral wrist extensors were similar for all conditions (Table 1). Ipsilateral reflex responses (not shown) were similar in all conditions in both arms and did not differ from results shown previously (Dietz et al. 2015; Schrafl‐Altermatt and Dietz 2016a).

**Table 1:** Latencies of reflex responses [msec]

	Bottle	Saw	Nut
Moving hand			
N2	86.92 ± 12.81	78.92 ± 14.08	86.72 ± 13.08
P2	149.44 ± 30.40	146.56 ± 30.55	135.13 ± 21.55
Holding hand			
N2	90.54 ± 20.41	82.05 ± 9.82	N/A
P2	148.67 ± 25.02	134.87 ± 23.45	N/A

The presence of EMG reflex responses was dependant on the task and the stimulated side. In Figure 3B the percentage of participants is presented who showed a contralateral reflex response in the respective tasks. In line with the recordings shown in Figure 2, the presence of reflex responses in the moving arm following stimulation of the holding arm is lowest in the saw condition. However, still 60% of the participants showed a reflex response in this condition. Eighty percent and 87% of the participants showed reflexes in the bottle and the nut condition, respectively. Following stimulation of the moving hand, the presence of EMG reflexes was highest in the saw condition (87%) followed by the bottle condition (80%). In the nut condition, only 27% of all participants showed a reflex response in the extensor muscles of the holding hand.

The duration of the EMG reflex responses (Fig. 3C) was similar in the bottle (89.2 ± 19.3 msec) and in the saw (91.7 ± 18.2 msec) task. However, it was significantly shorter (*P* = 0.0019) in the nut task (64.1 ± 14.4 msec). This data is an average over responses following stimulation of both the moving and of the holding hand, as there was no difference between the two conditions.

### Kinematic analyses

**Figure 4:**
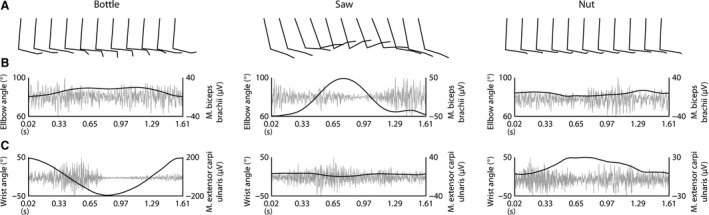
Motion capture. Kinematic data of the dominant moving arm was recorded in one participant and is displayed for the three cooperative hand movement conditions, that is, bottle, saw and nut. (A) Stick diagrams of the upper limb composed of three segments (upper arm, lower arm and hand) over one movement cycle are shown. One stick diagram for each tenth of the cycle is displayed. (B) Mean values of the elbow angle (black) and the electromyographic signal (EMG) recorded in the biceps muscle (gray) are shown over one movement cycle. (C) Mean values of the wrist angle (black) and the EMG recorded in the wrist extensor muscle (gray) are shown over one movement cycle. Note the different calibration for the EMG in the different conditions.

Figure 4 shows the motion capture analysis performed in one participant. In Figure 4A, the sagittal views of the dominant upper limb are displayed composed of upper and lower arm segments as well as the hand. Additionally, the elbow angle combined with EMG activity of the biceps (Fig. 4B) as well as the wrist angle combined with forearm extensor (Fig. 4D) EMG for all three movement conditions of the dominant arm of this participant. EMG of triceps and wrist flexor is not shown. The activity in these muscles was modulated in a phase‐dependent manner to a similar extent as biceps and wrist extensors.

The movement cycle time was the same in all conditions (in this case slightly longer than instructed: 1.6s instead of 1.5s). However, during the nut condition, there was a movement pause in the middle and the end/beginning of each cycle reflected by a plateau in both elbow and wrist angle. This means that although one entire cycle had the same duration, the actual moving time was shorter during the nut compared to saw and bottle conditions. In the latter conditions the moving time was the same, that is, it lasted over the entire movement cycle. The elbow angle had the widest range in the saw condition (60° to 100°), while it stayed quite stable in the bottle (80° to 88°) and the nut (80° to 86°) conditions. The range of wrist angle movement amplitudes was largest during the bottle task (−50° to 50°). It was smaller during the nut (10° to 50°) and smallest during the saw (2° to 10°) condition.

### Ipsi‐ and contralateral SSEP recordings

**Figure 5:**
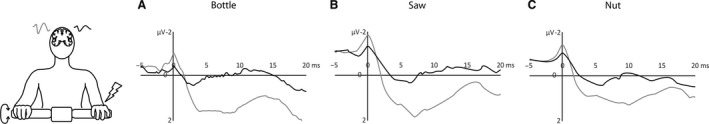
Somatosensory evoked potentials (SSEPs): Grand averages. Grand averages of SSEP of all (n = 15) participants. Ipsilateral (black) and contralateral (gray) SSEPs were recorded over hand areas of the primary somatosensory cortical areas following stimulation of the non‐dominant holding hand during the three movement conditions. The signals were time‐normalized, that is, the N20 peak of each participant's potential was set to zero before calculation of grand averages.

**Figure 6:**
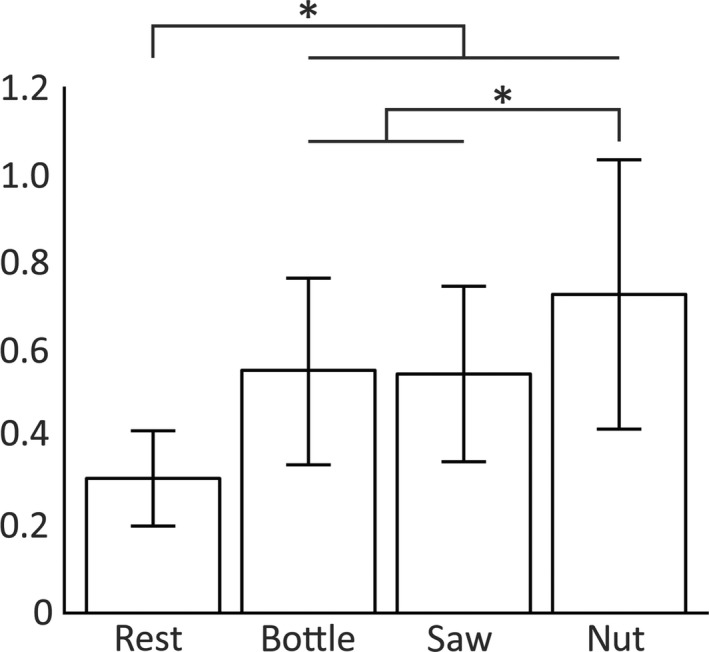
Ipsi‐ and contralateral somatosensory evoked potentials (SSEPs): Amplitude ratios. Amplitude ratios calculated for all participants (*n* = 15) are shown for the three cooperative movement conditions and for the resting condition. The ratio was calculated for each participant by division of the amplitude of the ipsilateral potential by the amplitude of the contralateral potential. Error bars: standard deviation; *P < 0.05.

**Figure 7:**
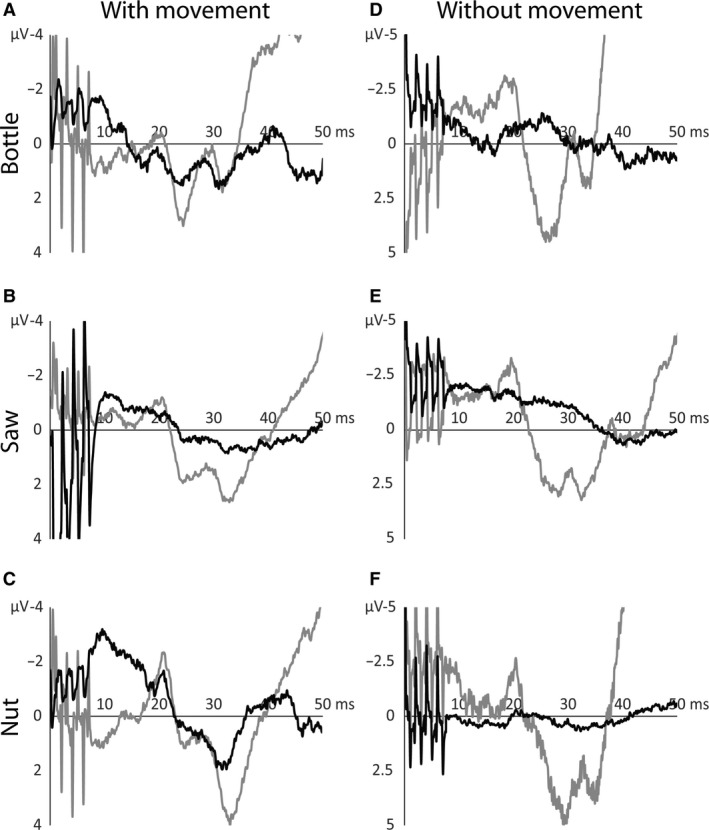
Comparison of somatosensory evoked potentials (SSEPs) during moving and static conditions. Averaged electro‐encephalic traces of one subject. (A‐C): SSEPs evoked during the three movement conditions. (D–F): SSEPs evoked during static holding of the device with the same handles as in the corresponding movement condition. Ipsilateral potentials (black lines) show higher amplitudes and are modulated, that is, highest nut condition, in the movement conditions compared to. The smaller potentials in the nonmoving conditions are similar in all three conditions. Contralateral potentials (gray lines) are higher in the nonmoving conditions. They are neither modulated in the moving nor in the nonmoving conditions.

Figure 5 shows the grand averages of time‐normalized ipsi‐ and contralateral SSEPs during the three cooperative movement tasks. Only SSEPs elicited by stimulation of the holding hand are shown as no major differences were observed between potentials following stimulation of the holding and the moving hand. Both ipsi‐ and contralateral potentials were elicited in the resting condition (not shown) as well as in all three movement tasks. The amplitude ratio shown in Figure 6 was significantly higher during all three cooperative hand movements compared to resting (bottle: *P* = 0.000125; saw: *P* = 0.000015; nut: *P* = 0.000005). The amplitude ratio in the nut condition was again significantly higher compared to the ratios in saw (*P* = 0.045) and bottle (*P* = 0.018) conditions, that is, the ipsilateral SSEP amplitude was only slightly smaller compared to the contralateral amplitude. Figure 7 shows SSEP traces of one participant during the three moving conditions (A‐C) as well as during the three additional nonmoving conditions (D‐F). It is shown that contralateral SSEPs are smaller during movement but still clearly defined. Ipsilateral SSEPs, on the other hand, are increased in amplitude during cooperative hand movements. The modulation of ipsilateral SSEPs can only be observed during the different cooperative movements and not by simply changing the position of the hands during the three nonmoving conditions. Latencies, both N20 and P25, were equivalent for ipsi‐ and contralateral potentials in all conditions (Table 2).

**Table 2:** Latencies of somatosensory evoked potentials [msec]

	Rest	Bottle	Saw	Nut
Contralateral				
N20	19.08 ± 1.17	19.43 ± 1.77	19.76 ± 1.92	19.47 ± 1.91
P25	25.28 ± 3.04	25.22 ± 3.31	25.86 ± 3.27	26.11 ± 3.04
Ipsilateral				
N20	19.39 ± 1.30	19.92 ± 1.94	19.94 ± 2.20	19.98 ± 2.21
P25	24.21 ± 2.87	24.39 ± 2.66	25.47 ± 2.84	25.41 ± 3.09

## Discussion

The aim of the study was to explore the behavior of the neural coupling mechanism in different cooperative hand movement tasks. The chosen tasks simulated activities of daily living: opening a bottle or a jar, sawing wood or slicing bread and screwing a nut onto a bolt. During the experiment, the movements had to be performed in a supine position in order to minimize artifacts in the EEG recordings which is in contrast to performance of these tasks during daily living. However, the device was freely held in the air without supporting the arms on the bench which ensured similar muscle activation compared to upright task performance. Contralateral reflex responses during the bottle task were similar to those described previously which were recorded in a sitting position (Dietz et al. 2015). This indicates that the neural control of cooperative hand movements is not influenced by postural position. All three movement tasks showed the basic characteristics of neural coupling, that is, contralateral EMG reflex responses in the activated forearm muscles following unilateral ulnar nerve stimulation and an enhanced ipsi‐ to contralateral SSEP ratio compared to resting. Both of these aspects of neural coupling were task‐specifically modulated and thus varied in their appearance and expression.

### One control mechanism, different key components

So far, three aspects of the neural coupling involved in the control of cooperative hand movements have been demonstrated. First, a stronger activation of the S2 cortical areas is present during cooperative hand movements compared to other bimanual tasks (Dietz et al. 2015). It is known that each S2 cortical area receives afferent input from both hands (Lin and Forss 2002) indicating an involvement of ipsilateral hemispheres in movement control. Second, bilateral arm muscle reflex responses to unilateral electrical nerve stimulations (Dietz et al. 2015; Schrafl‐Altermatt and Dietz 2016a) are evidence for a task‐specific functional coupling of the two hands during cooperative movements. This is in line with the neural interlimb coupling in other functional tasks such as balancing (Dietz and Berger 1982) or walking (Dietz 2002; Dietz and Michel 2009; Kloter and Dietz 2012). Lastly, enhanced ipsilateral SSEP amplitudes during cooperative compared to non‐cooperative bimanual movements recorded over S1 (Schrafl‐Altermatt and Dietz 2014, 2016b). This enhancement might be associated with the extra‐activation of the S2 areas during cooperative tasks. S2 areas are anatomically connected to cortical S1 areas (Krubitzer and Kaas 1990) and thus might explain the high activity in both the ipsilateral S1 (SSEP)and S2 (fMRI) areas during cooperative hand tasks. These observations highlight the importance of a task‐specific processing of ipsilateral ascending input for a successful execution of goal‐directed cooperative hand movements. As analyzed in this study, both electrophysiological measures, that is, contralateral reflex responses and ipsilateral SSEP amplitudes were modulated by the different tasks in different ways.

### Fine cooperative movements – unbalanced neural coupling

The small cooperative movements (nut) studied here differed in the control from the well‐established bottle task with regard to both the SSEP and the reflex behavior. The SSEP amplitude ratio was highest during this fine movement task. This finding indicates a more global and less lateralized control of this movement task. This might be due to the fact that fine finger and hand movements are known to be under stronger cortical control than gross movements (Wiesendanger and Miles 1982; Lemon 2008).

In addition, a contralateral reflex response was only elicited in the moving, that is, dominant, hand extensors but not in the holding hand. This finding would be in line with a hemispherical asymmetry in the sensorimotor cortex towards the dominant hemisphere during an isometric bimanual cooperative task (Theorin and Johansson 2007). An alternative explanation might be that no contralateral EMG responses could be elicited during an isometric cooperative task (Dietz et al. 2015). In the nut condition the counteracting holding hand was more static compared to the gross movements. Thus, the stabilizing function of the holding hand during the fine movement might explain the lack of a reflex response from the moving to the holding hand.

### Gross cooperative movements –balanced neural coupling

The newly investigated cooperative task “saw” did not much differ in its neural control from the “bottle” task. In both tasks the SSEP amplitude ratio was higher compared to resting with ipsilateral amplitudes about half as high as contralateral amplitudes. Contralateral reflex responses were, on average, evenly elicited on both sides despite the kinematic differences of the holding and the moving hand. This suggests reciprocity in the neural control of movement performance on both sides although basic kinematic differences exist between the acting and holding hand in both tasks. This might be due to the fact that similar efforts were exerted by both hands in these tasks. This suggestion would be in line with the notion that cortical activity (especially S2) can be modulated by the exerted effort (Heuninckx et al. 2005; Goble et al. 2010). A slight difference between bottle and saw task existed in the prevalence of contralateral reflex responses. While the presence of EMG responses was the same in both arms during the bottle task, in the saw task more participants showed stronger reflex responses in the holding than the moving hand. This could be due to the lower background activity in the forearm muscles on the moving side during the saw task, leading to down‐regulated reflex activity (Forgaard et al. 2015). An alternative explanation might be that the same muscle groups were activated on both sides during bottle but different prime actors were active on each side during the saw movement. This difference might affect the involvement of ipsilateral descending pathways and, consequently, the expression of the reflex responses (Tazoe and Perez 2014).

### Task‐characteristics encoded in reflex behavior

The fine and gross movement tasks studied here differed in movement amplitude. This difference was reflected in the duration of the contralateral reflex responses but not in the appearance of ipsilateral SSEP. This indicates that physical properties of the respective cooperative movement task become encoded and consecutively expressed in the reflex behavior. This executory function of the reflex activity is assumed to take place under the control mechanism of neural coupling. This suggestion is in line with the observation of a cortical control of neural coupling of arms and legs during locomotor tasks (Haefeli et al. 2011). The adaptive reflex behavior during the different tasks suggests an online control of cooperative hand movements and not just a simple reaction to contralateral electrical stimulation. While also the SSEP were modulated by the different tasks, adaptive changes to physical task conditions such as movement duration and amplitude were not reflected in the SSEP signals. Nevertheless a cortical control of the reflex activity can be assumed to occur (Tanji et al. 2007).

## Conclusions

According to this study, the various cooperative hand movements that are required during activities of daily living are all based on a task‐specific neural control, that is, the neural coupling mechanism. This neural coupling involves the ipsilateral hemisphere and a functional coupling of upper limbs. This task‐specific neural control might have consequences for the rehabilitation of hand function after a stroke. In most poststroke participants the neural coupling is preserved from the unaffected to the affected limb (Schrafl‐Altermatt and Dietz 2016a). Through the involvement of the unaffected hemisphere in the movement control of the paretic hand, training various cooperative movements needed during ADL's may be beneficial and lead to an improved outcome of hand function. Besides the activation of the neural coupling mechanism, both fine and gross hand movements required in ADL's would be trained by such an approach.

## Conflict of Interest

The authors declare no competing financial interests.
